# TIM-4 orchestrates mitochondrial homeostasis to promote lung cancer progression *via* ANXA2/PI3K/AKT/OPA1 axis

**DOI:** 10.1038/s41419-023-05678-3

**Published:** 2023-02-20

**Authors:** Yuzhen Wang, Yingchun Wang, Wen Liu, Lu Ding, Xiaodi Zhang, Bo Wang, Zheng Tong, Xuetian Yue, Chunyang Li, Liyun Xu, Zhuanchang Wu, Xiaohong Liang, Chunhong Ma, Lifen Gao

**Affiliations:** 1 Key Laboratory for Experimental Teratology of Ministry of Education, Shandong Key Laboratory of Infection and Immunity, and Department of Immunology, School of Basic Medical Sciences, Cheeloo College of Medicine, Shandong University, Jinan, Shandong 250012 P. R. China; 2 Key Laboratory for Experimental Teratology of Ministry of Education, Department of Cell Biology, School of Basic Medical Sciences, Cheeloo College of Medicine, Shandong University, Jinan, Shandong 250012 P. R. China; 3 Key Laboratory for Experimental Teratology of Ministry of Education, Department of Histology and Embryology, School of Basic Medical Sciences, Cheeloo College of Medicine, Shandong University, Jinan, Shandong 250012 P. R. China; 4 Cell and Molecular Biology Laboratory, Zhoushan Hospital, Zhoushan, Zhejiang 316000 P. R. China

**Keywords:** Cancer metabolism, Non-small-cell lung cancer

## Abstract

Mitochondrial function and homeostasis are critical to the proliferation of lung cancer cells. T-cell immunoglobulin and mucin domain-containing molecule 4 (TIM-4) promotes the development and progression of lung cancer. However, the role of TIM-4 in mitochondria homeostasis in tumor cells remains completely unknown. In this study, we found that TIM-4 promoted growth and proliferation of lung cancer cells by the oxidative phosphorylation (OXPHOS) pathway. Consistently, inhibition of OXPHOS reversed TIM-4-induced proliferation of lung cancer cells. Notably, TIM-4 promoted mitochondrial fusion via enhancing L-OPA1 protein expression. Mechanistically, TIM-4 regulated protein of L-OPA1 through the PI3K/AKT pathway, and TIM-4 interacted with ANXA2 to promote the activation of PI3K/AKT signaling. Collectively, TIM-4 promotes oxidative phosphorylation of lung cancer cells to accelerate tumor progress *via* ANXA2/PI3K/AKT/OPA1 axis, which sheds significant new lights on the potential role of TIM-4 in regulating tumor cell metabolism.

## Introduction

Lung cancer is the leading cause of cancer-related deaths worldwide. 85% of lung cancer belongs to non-small cell lung cancer (NSCLC). With limited curative treatment options, NSCLC can change their growth characteristics to induce therapeutic resistance [1, 2]. Metabolic alterations distinguish lung cancer cells from healthy cells, which is recognized as one of the 10 hallmarks of cancer [3]. Altered energy production pathways help cancer cells to sustain high proliferative rates, even in a hostile environment characterized by hypoxia and insufficient nutrient supply [4]. Therefore targeted energy metabolism might be a novel approach for tumor therapy [5].

In the decades, Otto Warburg first proposed that tumor cells have a high glycolysis rate even under oxygen-rich conditions, a phenomenon known as aerobic glycolysis or the Warburg effect. Most tumor cells show a metabolic state with low levels of oxidative phosphorylation (OXPHOS) and display complete mitochondrial dysfunction [6, 7]. Consistently, many kinds of cancers, including melanoma and kidney cancer, are characterized by down-regulation of OXPHOS metabolism, accompanied by increased intermediate metabolites and end products produced by glycolysis [8]. However, increasing evidences indicate that tumor cells can reprogram metabolism to ensure cell proliferation underlying the environment [9, 10]. Recent studies show that glycolysis and OXPHOS can synergistically promote the development of colorectal cancer [11]. Notably, OXPHOS plays a critical role in energy metabolism of lung cancer cells [5, 12]. Currently, OXPHOS and electron transport chain as targets for cancer therapy have attracted the attention of many researchers [13, 14].

T-cell immunoglobulin and mucin domain-containing molecule 4 (TIM-4), is one of type I membrane protein consisting of a signal sequence followed by an immunoglobulin variable region (IgV)-like domain, a mucin-like domain, a transmembrane region and an intracellular tail [15]. TIM-4 is highly expressed in human and mouse macrophages and dendritic cells, regulating innate and adaptive immunity. As a receptor for phosphatidylserine recognition, TIM-4 is essential for the effective clearance of apoptotic cells and the prevention of autoimmunity [16, 17]. Recently, TIM-4 emerges as a novel immune checkpoint on antigen-presenting cells. TIM-4^+^ macrophages impair proliferation and function of CD8^+^ T cells [18], indicating the key role of TIM-4 in anti-tumor response. Interestingly, TIM-4 is found to be highly expressed in a variety of tumors including lung cancer [19]. TIM-4 promotes the growth of colorectal cancer by activating angiogenesis and recruiting tumor-associated macrophages (TAMs) *via* the PI3K/AKT/mTOR signaling pathway [20], however, the exact mechanism remains unknown. Previously, we found that TIM-4 could promote the growth of NSCLC in a Arg-Gly-Asp (RGD) motif-dependent manner and also participated in lung cancer progression by responding to IL-6 signaling [19, 21]. It is reported that TIM-4^+^ TAMs exhibit high levels of OXPHOS and can alleviate oxidative stress by regulating mitochondrial autophagy [22]. However, the role of TIM-4 in tumor cell metabolism remains completely unclear.

In this study, we found that overexpression of TIM-4 increased the OXPHOS level and promoted mitochondrial function of lung cancer cells. We further found that TIM-4 regulated mitochondrial morphology and kinetic balance of lung cancer cells by upregulating protein expression of L-OPA1, making mitochondria tend to be fusion. Mechanistically, ANXA2/PI3K/AKT signaling pathway was responsible for TIM-4 mediated regulation of mitochondrial function in lung cancer cells. Overall, this work reveals a previously unidentified role for TIM-4 in metabolic reprogramming of lung cancer cells by orchestrating mitochondrial homeostasis, providing a novel potential target for lung cancer therapy.

## Results

### TIM-4 enhances the OXPHOS level of lung cancer cells

**Fig. 1:**
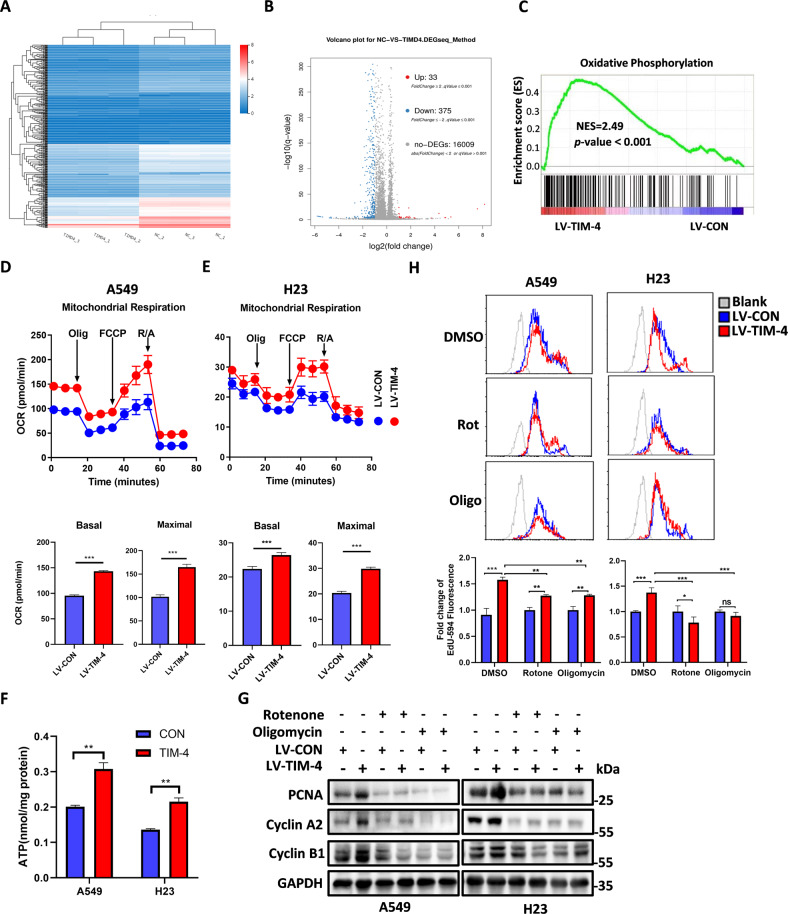
TIM-4 overexpression enhances oxidative phosphorylation level of lung cancer cells. **A**, **B** A549 cells overexpressing TIM-4 and control cells were analyzed by RNA-Seq, and cluster analysis and differentially expressed genes (false discovery rate [FDR] < 0.05, fold change ≥2) were visualized in a heatmap and volcano figure. **C** RNA-Seq analysis in A549 cells. GSEA was used to analyze the OXPHOS-related genes with differential mRNA levels. Normalized enrichment score (NES), *P*-value <0.05 is considered significant. **D**, **E** Mitochondrial oxygen consumption rate (OCR), mitochondrial basal respiration and maximal respiration of A549 cells and H23 cells. **F** ATP production in A549 and H23 cells overexpressing TIM-4 and the control cells. **G**, **H** A549 and H23 cells overexpressing TIM-4 and control cells were treated with rotenone (Rot) (0.5 μM) and oligomycin (Oligo) (2 μM) for 48 h, and PCNA, Cyclin A2, and Cyclin B1 expression were analyzed by western blotting, while Edu staining was measured by flow cytometry assay. Three independent experiments were conducted for each result and error bars represent SEM per group in one experiment. Data were analyzed using Student’s *t* test (two-tailed unpaired *t* test) for (**D**–**F**) and (**H**). ns means non-significance; *P < 0.05; **P < 0.01; ***P < 0.001.

Our previous work has shown that TIM-4 promotes the proliferation of lung cancer cells [19]. In order to further explore the mechanism of TIM-4 promoting lung cancer growth, we constructed stable cell lines A549-LV-TIM-4 and H23-LV-TIM-4 that overexpressed TIM-4 (Fig. S1A, B). Then A549-LV-TIM-4 and corresponding control cells were used to perform RNA sequencing (Fig. 1A, Fig. S1C). Data analysis showed that TIM-4 overexpression resulted in different expression of 408 genes (log 2 FC > 1) (Fig. 1B). The gene set enrichment analysis (GSEA) showed a significant enrichment of differentially expressed gene signatures in the OXPHOS pathway in TIM-4 overexpression group (Fig. 1C), indicating that TIM-4 might increase the metabolic level of lung cancer cells. We further detected the OXPHOS process of A549 cells using O2K energy metabolism system, and the results showed that TIM-4 significantly increased the OXPHOS level of A549 cells under different substrate stimulation (Fig. S1D, E). In addition, seahorse energy detection system was also used to detect oxygen consumption (OCR) of A549 and H23 cells, and the results showed that the basal OCR and maximum OCR in the TIM-4 overexpression group were higher than those in the control group (Fig. 1D, E). Collectively, these results reveal that TIM-4 enhances the OXPHOS level of lung cancer cells.

It is well known that OXPHOS takes place in mitochondria whereby cells use carbon fuels and oxygen to generate ATP. The mitochondrial respiratory chain consists of complexes I–V. The transport of electrons within NADH-ubiquinone oxidoreductase (complex I), ubiquinol-cytochrome c oxidoreductase (complex III), and cytochrome c oxidase (complex IV) is linked to proton pumping across the inner mitochondrial membrane (IMM), generating an electrochemical gradient (Δp) that is coupled to ATP production at ATP synthase (complex V) [23, 24]. In accordance with the modulation of TIM-4 on OXPHOS, we found that TIM-4 overexpression increased the ATP level of lung cancer cells (Fig. 1F). In order to further verify whether TIM-4 promotes the proliferation of lung cancer cells by enhancing OXPHOS, lung cancer cell lines A549 and H23 were treated with rotenone and oligomycin, inhibitors of complex I and complex V [25], respectively. In consistence with previous report, TIM-4 overexpression promoted proliferating cell nuclear antigen (PCNA), Cyclin A2, Cyclin B1 expression in lung cancer cells. However, rotenone and oligomycin largely eliminated PCNA, Cyclin A2, Cyclin B1 expression induced by TIM-4 in A549 and H23 cells (Fig. 1G). In addition, EdU staining of A549 and H23 cells was assayed by flow cytometry, and the results showed that rotenone and oligomycin greatly decreased TIM-4 promoted EdU incorporation in lung cancer cells (Fig. 1H). Thus, TIM-4 promotes the proliferation of lung cancer cells largely by increasing the OXPHOS levels.

### TIM-4 improves mitochondrial activity and fitness of lung cancer cells

**Fig. 2:**
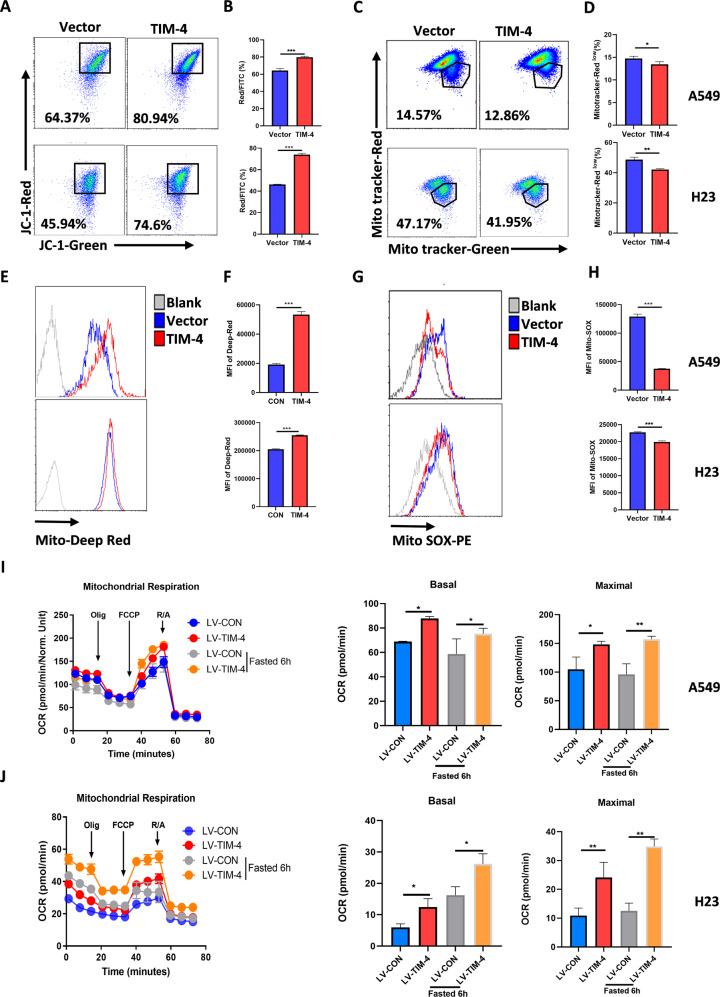
TIM-4 enhances mitochondrial activity and fitness of lung cancer cells. **A**, **B** Mitochondrial membrane potential (JC-1 aggregates) in A549 and H23 cells were accessed by the JC-1 assay kit according to the manufacture’s instruction. **C**, **D** Flow cytometry dot plots showed the percentage of depolarized mitochondria in A549 and H23 cell lines transfected with LV-CON or LV-TIM-4. **E**, **F** Mitochondria fitness was tested with Mito-tracker Deep Red. **G**, **H** Mitochondrial ROS levels of LV-A549 cells and LV-H23 cells were accessed by assay kit. **I**, **J** OCR, mitochondrial basal respiration and maximal respiration of LV-A549 cells and LV-H23 cells after fasted (Glucose:1 mM). Three independent experiments were conducted for each result and error bars represent SEM per group in one experiment. Data were analyzed using Student’s *t* test (two-tailed unpaired *t* test). ns means non-significance; *P < 0.05; **P < 0.01; ***P < 0.001.

**Fig. 3:**
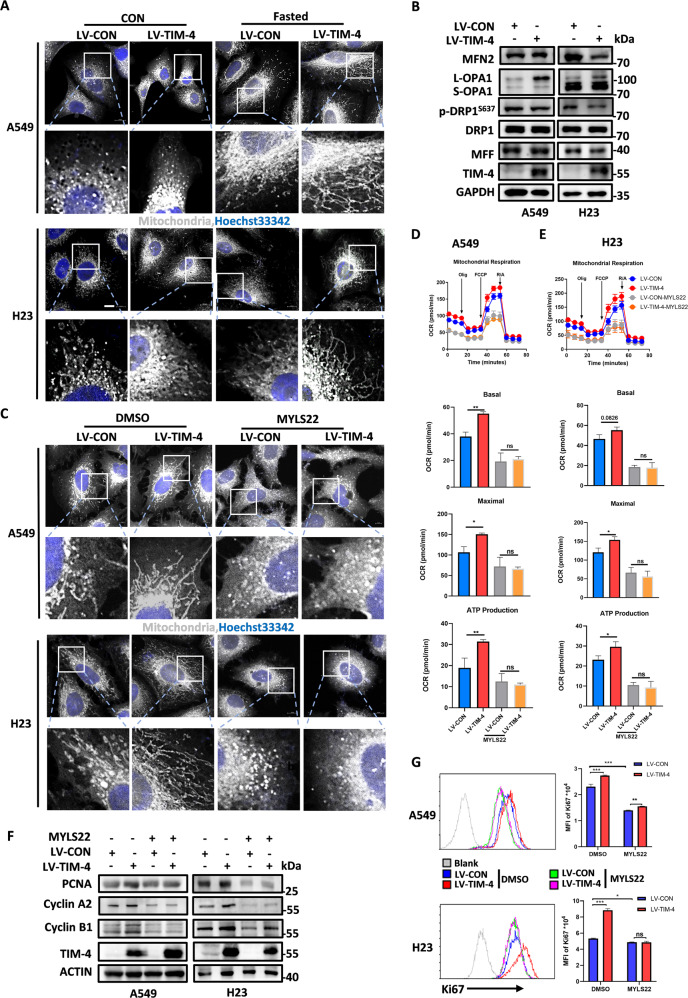
TIM-4 promotes mitochondrial fusion and function by increasing the level of L-OPA1. **A** Representative confocal micrographs of mitochondrial morphology in A549 and H23 cells. Mitochondria were visualized by anti-TOM20 immunostaining (gray), Hoechst 33342 labels nuclei (blue). The scale bar represents 10 μm. **B** Levels of proteins involved in mitochondrial dynamics (MFN2, OPA1, p-DRP1, DRP1, and MFF) were detected by western blotting in A549 and H23 cells. **C** A549 and H23 cells transfected with LV-CON and LV-TIM-4 were treated with MYLS22 (50 μM) for 24 h, then cells were subjected to anti-TOM20 (gray), DAPI Hoechst 33342 staining (blue), scale bar = 10 μm. **D**, **E** OCR, mitochondrial basal respiration and maximal respiration of A549 and H23 cells treated with inhibitor of OPA1 (MYLS22: 50 μM) for 24 h. **F** The expression levels of cell proliferation marker PCNA and cell cycle-related proteins including Cyclin A2, Cyclin B1 were determined by western blotting in A549 and H23 cells treated with MYLS22 for 24 h. **G** Flow cytometry histograms showing the level of Ki67 in lung cancer cell lines treated with MYLS22 for 24 h. Three independent experiments were conducted for each result and error bars represent SEM per group in one experiment. Data were analyzed using Student’s *t* test (two-tailed unpaired *t* test) for (**D**), (**E**), and (**G**). ns means non-significance; *P < 0.05; **P < 0.01; ***P < 0.001.

The healthy status of mitochondria is crucial for the survival of cells [5]. Since TIM-4 enhances the OXPHOS level of lung cancer cells, we wonder whether TIM-4 affects the activity and fitness of mitochondria. Therefore, we tested the mitochondria membrane potential (ΔΨm) of lung cancer cell lines A549 and H23 by JC-1 mitochondrial membrane potential assay kit. As shown in Fig. 2A, B, TIM-4 overexpression significantly enhanced the mitochondrial membrane potential of A549 and H23 cells. Then A549 and H23 cells were stained with Mito-Tracker Green/Red to detect mitochondrial depolarization [26], and the results showed that TIM-4 overexpression reduced the numbers of depolarized mitochondria (Fig. 2C, D). Mito-Tracker Deep Red could label healthy mitochondria. As predicted, the cells in the TIM-4 overexpression group had more healthy mitochondria (Fig. 3E, F). Since the level of mitochondrial ROS (mtROS) is crucial for the health and function maintenance of mitochondria [27], we detected the mtROS level of A549 and H23 cells. We found that the overexpression of TIM-4 significantly reduced the mtROS level of lung cancer cells (Fig. 2G, H). Emerging evidence shows that short time fasting can improve the adaptability of mitochondria, and the adaptability shows the mitochondrial activity of the cell under healthy situation [28, 29]. Thus we treated cells with glucose limitation (1 mM glucose). The results manifested that adaptability of mitochondria was significantly higher in TIM-4 overexpression group than that of control group (Fig. 2I, J). Altogether, TIM-4 enhances mitochondria activity and maintains mitochondria fitness of lung cancer cells.

### TIM-4 promotes mitochondrial fusion by increasing the level of L-OPA1

Mitochondria are highly dynamic organelles undergoing coordinated cycles of fission and fusion, referred as ‘mitochondrial dynamics’. Their transient and rapid morphological adaptations are crucial for mitochondrial quality control, and imbalanced mitochondrial dynamics are associated with a range of diseases characterized by impaired mitochondrial function and increased cell death [30]. We used immunofluorescence staining technology of TOM20 to mark the mitochondria of A549 and H23 cells to evaluate the effects of TIM-4 on mitochondrial dynamics. The results showed that the morphology of mitochondria in the cells of the TIM-4 overexpression group was longer and more concentrated, which was more obvious under the condition of low-glucose stimulation compared with the control group (Fig. 3A). Then we further detected the protein levels of OPA1, MFN2 (mitochondrial fusion-related molecules), p-DRP1, DRP1 (dynamin-related protein 1) and MFF (mitochondrial fission related molecules) respectively, and the results showed that the protein levels of L-OPA1 were significantly increased in the TIM-4 overexpression group (Fig. 3B). However, the expression of OPA1 at the transcriptional level showed no significant difference between the TIM-4 overexpression and control group (Fig. S2A, B). Consistent with the point that OPA1 is a key regulator of mitochondrial crest remodeling [31], we found that TIM-4 significantly increased the density of mitochondrial crest using transmission electron microscopy (Fig. S2C).

To identify the key role of OPA1 in TIM-4-promoted mitochondrial fusion and function, MYLS22, a specific inhibitor of OPA1 [32], was included to treat A549 and H23 cells, and then the morphology of mitochondria and OXPHOS were detected. As expected, MYLS22 treatment caused mitochondria fragmented in control group, while TIM-4 overexpression-induced mitochondrial fusion no longer existed under MYLS22 stimulation (Fig. 3C). Consistently, MYLS22 significantly reduced the level of OXPHOS induced by TIM-4 overexpression (Fig. 3D, E). siRNA targeting OPA1 also reversed the promotion of OXPHOS by TIM-4 in lung cancer cells (Fig. S2D–F). As expected, siRNA targeting OPA1 also reversed the promotion of mitochondrial activity induced by TIM-4 overexpression in lung cancer cells, including the increase in mitochondrial membrane potential (Fig. S2G, H), and the differences in the numbers of damaged and healthy mitochondria (Fig. S2I–L). In addition, MYLS22 effectively attenuated the increased expression of PCNA and cell cycle molecules (Cyclin A2, Cyclin B1) (Fig. 3F), as well as Ki67 induced by TIM-4 (Fig. 3G). These findings suggest that OPA1 is involved in TIM-4-promoted lung cancer progression.

### TIM-4 regulates mitochondrial function through the PI3K/AKT signaling pathway

**Fig. 4:**
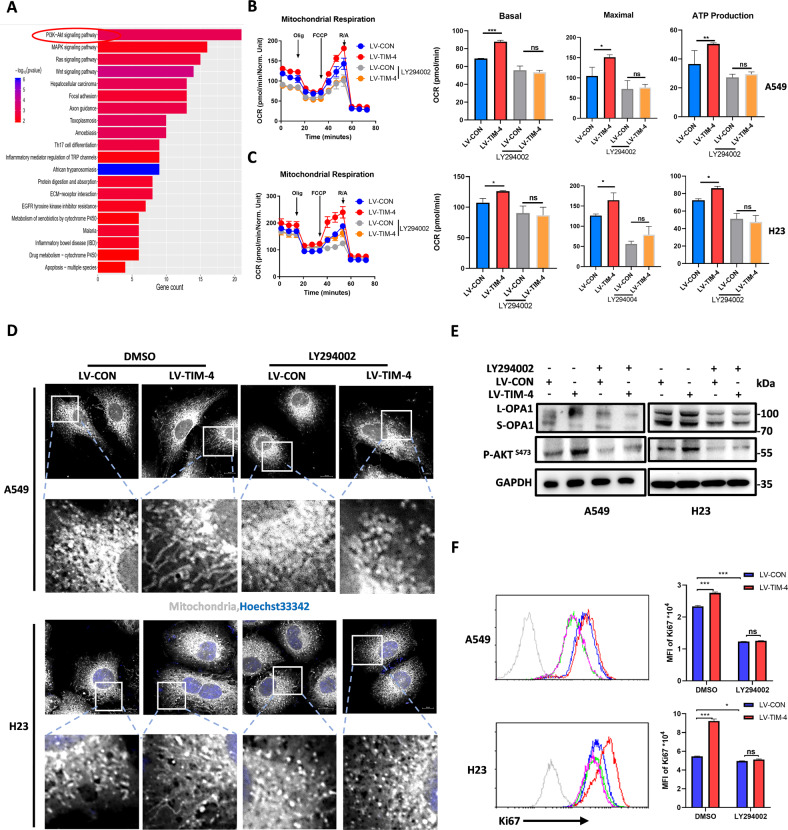
TIM-4 regulates mitochondrial function through the PI3K/AKT signaling pathway. **A** Kyoto Encyclopedia of Gene and Genomes (KEGG) analysis showed top enriched gene sets based on RNA-seq data. **B**, **C** OCR, mitochondrial basal respiration and maximal respiration of A549 and H23 cells treated with inhibitor of AKT (LY294002, 50 μM) for 24 h. **D** Immunofluorescent staining of A549 and H23 cells were treated with the indicated inhibitor (LY294002, 50 μM) for 24 h and mitochondrial phenotype was assessed by TOM20 staining. The scale bar represents 10 μm. **E** OPA1 levels and phosphorylation of AKT were assessed by immunoblotting in lung cancer cell lines treated with DMSO or LY294002 (50 μM) for 24 h. **F** Flow cytometry histograms showing the level of Ki67 in lung cancer cell lines treated with DMSO or LY294002 for 24 h. Three independent experiments were conducted for each result and error bars represent SEM per group in one experiment. Data were analyzed using Student’s *t* test (two-tailed unpaired *t* test) for (**B**), (**C**), and (**F**). ns means non-significance; *P < 0.05; **P < 0.01; ***P < 0.001.

In order to further explore the mechanism of TIM-4 regulating mitochondrial function in lung cancer, transcriptome sequencing was further analyzed. We found that the PI3K/AKT signaling pathway was significantly enriched in the TIM-4 overexpression group (Fig. 4A). PI3K/AKT signaling pathway contributes to the occurrence and progression of various tumors and also involved in mitochondrial metabolism [33]. To further verify whether TIM-4 regulates mitochondrial function through the PI3K/AKT signaling pathway, we treated lung cancer cells with LY294002, an inhibitor of AKT [34], and the OXPHOS ability was assayed. The results showed that inhibition of PI3K/AKT signaling significantly attenuated the upregulation of OXPHOS induced by TIM-4 in both A549 and H23 cells (Fig. 4B, C). We also found that the mitochondria of lung cancer cells in LY294002 treatment group was significantly shorter than that of control group, and LY294002 abolished the mitochondrial fusion caused by TIM-4 overexpression (Fig. 4D). In addition, LY294002 significantly reduced L-OPA1 levels, even in the TIM-4 overexpression group (Fig. 4E). To further elucidate the role of PI3K/AKT signaling pathway in TIM-4 enhanced mitochondrial function in lung cancer cells, we used siRNA to interfere with AKT1 expression (Fig. S3A). Similar to the effect of inhibitor LY294002, TIM-4 promoted mitochondrial function and activity of lung cancer cells were reduced, including OCR (Fig. S3B, C), mitochondrial membrane potential (Fig. S3D, E), proportions of depolarized mitochondria (Fig. S3F, G), and numbers of healthy mitochondria (Fig. S3H, I). Moreover, LY294002 stimulation reduced TIM-4 promoted Ki67 levels in both A549 and H23 cells (Fig. 4F). Overall, our data reveal that PI3K/AKT signaling plays a crucial role in TIM-4 mediated regulation of mitochondria activity and proliferation of lung cancer cells.

### TIM-4 interacts with ANXA2 to regulate the PI3K/AKT signaling pathway

**Fig. 5:**
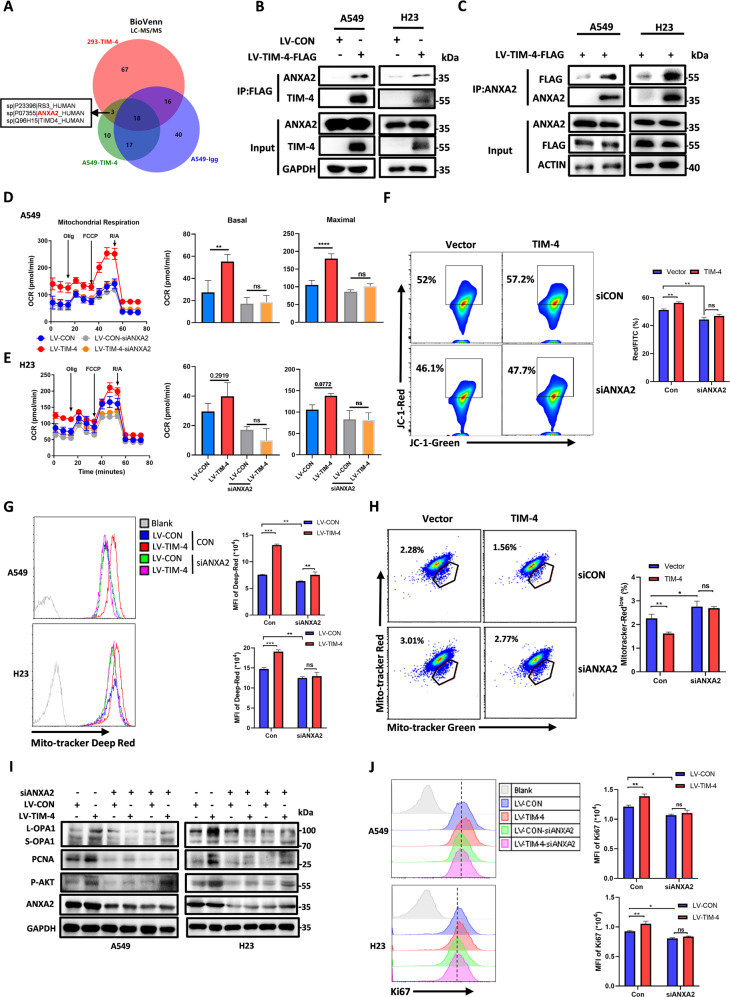
TIM-4 interacts with ANXA2 to regulate the PI3K/AKT signaling pathway and mitochondrial function. **A** Venn diagram depicting the comparison of proteins identified in A549-LV-CON, A549-LV-TIM-4, and HEK-293 cells. The shared proteins were excluded from the differential lists. **B**, **C** Co-immunoprecipitation (Co-IP) assays were performed in A549 and H23 cells overexpressing TIM-4 or control. **D**, **E** OCR, mitochondrial basal respiration and maximal respiration of A549 cells and H23 cells transfected with siRNA of ANXA2 or control. **F** Mitochondrial membrane potential (JC-1 aggregates) in A549 cells transfected with siRNA targeting ANXA2 were accessed by the JC-1 assay kit according to the manufacture’s instruction. **G** Mitochondria fitness was tested with Mito-tracker Deep Red. **H** Flow cytometry dot plots showed the percentage of depolarized mitochondria in A549 cell lines transfected with siRNA targeting ANXA2. **I** Western blotting analysis of OPA1, p-AKT, PCNA, and ANXA2 expression in siANXA2-transfected lung cancer cell lines. **J** Flow cytometry histograms showing the level of Ki67 in lung cancer cell lines transfected with siRNA targeting ANXA2. Three independent experiments were conducted for each result and error bars represent SEM per group in one experiment. Data were analyzed using Student’s *t* test (two-tailed unpaired *t* test) for (**D**), (**E**), (**F**–**H**), and (**J**). ns means non-significance; *P < 0.05; **P < 0.01; ***P < 0.001.

To identify how TIM-4 promotes PI3K/AKT activation, TIM-4 interactors were coimmunoprecipitated (Co-IP) and subjected to LC/MS-MS analysis in A549 and HEK293 cells. A total of 3 proteins including annexin A2 (ANXA2), located on the cell membrane, were identified and caught our attention (Fig. 5A and Fig. S4A). The annexin family is a new family of calcium-binding proteins which bind phospholipids in a calcium-dependent manner [35, 36]. ANXA2 has been reported to promote tumor proliferation and metastasis as well as AKT activation [37–40]. Mutual Co-IP confirmed that TIM-4 physically interacted with ANXA2 in the two lung cancer cell lines (Fig. 5B, C). Next, we found that TIM-4 had no significant effect on the transcriptional level (Fig. S4B) and the protein expression of ANXA2 (Fig. 5B). We then investigated whether ANXA2 participates in TIM-4 mediated regulation of mitochondrial function in lung cancer cells. We found that ANXA2 knockdown inhibited TIM-4 promoted OXPHOS including the reduction of OCR (Fig. 5D, E) and (ΔΨm) (Fig. 5F), followed by an impairment of mitochondria fitness (Fig. 5G, H) in A549 and H23 cell lines, indicating that TIM-4 regulates OXPHOS *via* ANXA2.

It has been reported that ANXA2 can regulate the activation of PI3K/AKT signaling. Consistently, we found that interference of ANXA2 significantly inhibited AKT phosphorylation induced by TIM-4 (Fig. 5I). Furthermore, ANXA2 knockdown also reduced the protein level of mitochondrial fusion-related molecule L-OPA1 and the expression level of proliferation marker PCNA and Ki67 induced by TIM-4 (Fig. 5I, J). Therefore, TIM-4 promotes mitochondrial function and participates in lung cancer proliferation *via* ANXA2/PI3K/AKT axis.

### Patients with high expression of TIM-4 show higher levels of OXPHOS in lung cancer tissues

**Fig. 6:**
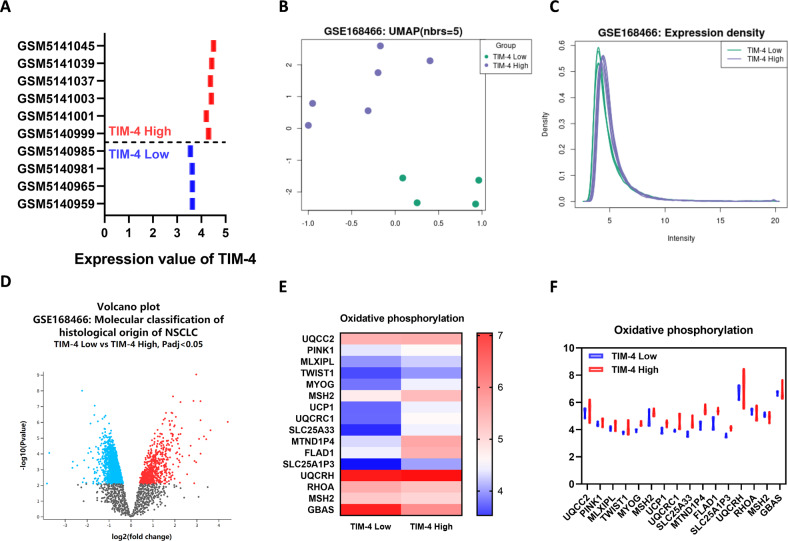
OXPHOS-related genes are upregulated in TIM-4^High^ patients. **A** Expression value of TIM-4 in tumor samples of 10 patients with NSCLC (TIM-4^High^, *n* = 6; TIM-4^Low^, *n* = 4) (Gene Expression Omnibus accession no. GSE168466). **B** Uniform Manifold Approximation and Projection (UMAP) showed significant differences between the two groups. **C** Normalization of the data analyzed by GEO2R. **D** Volcano plot of differentially expressed genes in tumor samples of 10 patients with NSCLC. **E** Heatmap of oxidative phosphorylation related genes expression in two groups. **F** Expression values of OXPHOS-related genes in TIM-4^High^ and TIM-4^Low^ patients.

To further clarify whether TIM-4 can enhance mitochondrial OXPHOS in lung cancer patients, a dataset with TIM-4 expression profiling by array in the GEO database was included. Ten NSCLC samples including TIM-4 low expression group (TIM-4^Low^) and TIM-4 high expression group (TIM-4^High^) were analyzed and screened (Fig. 6A). GEO2R analysis was performed according to the TIM-4 expression profiling in two groups of samples. Uniform Manifold Approximation and Projection (UMAP) showed significant differences between the two groups (Fig. 6B). The data normalization was shown in Fig. 6C. A volcano plot displayed statistical significance (−log10 P value) versus magnitude of change (log2 fold change) and visualized differentially expressed genes between TIM-4^Low^ group and TIM-4^High^ group (Fig. 6D). Among the differentially expressed genes, most of OXPHOS-related genes were upregulated in TIM-4^High^ patients (Fig. 6E, F). Together, the data suggest that TIM-4 does enhance mitochondrial OXPHOS of lung cancer tissues, supporting the role of TIM-4 in metabolism reprogramming of lung cancer.

## Discussion

**Fig. 7:**
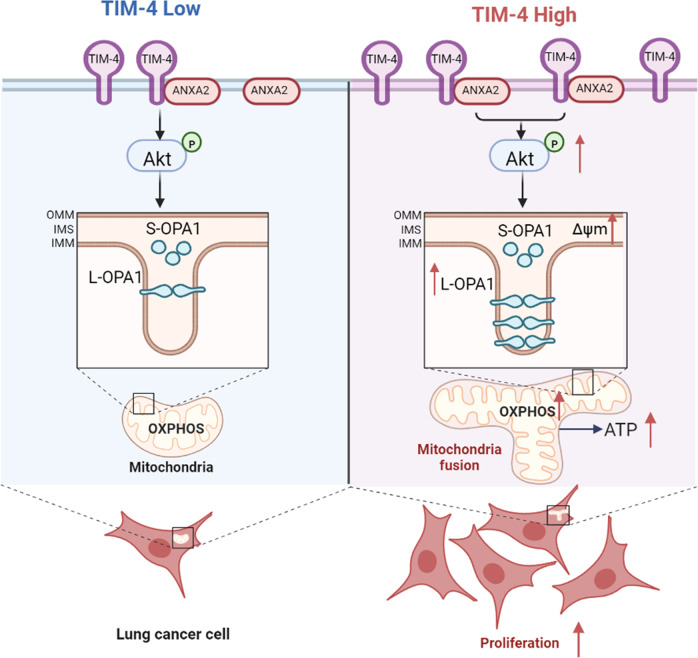
Schematic illustration for TIM-4 orchestrating mitochondrial homeostasis *via* ANXA2/PI3K/AKT/OPA1 axis in lung cancer. TIM-4 interacts with ANXA2 to activate PI3K/AKT signaling and promotes L-OPA1 expression. When TIM-4 expression is elevated, AKT activation is enhanced to maintain L-OPA1 levels and enhance mitochondrial fitness. The maintenance of L-OPA1 leads to a more fusion state of mitochondria, which leads to increased OXPHOS levels, thus promoting lung cancer cell proliferation.

Metabolic reprogramming plays a crucial role in the occurrence and development of tumors. Meanwhile, the change of metabolic pattern can affect the growth and survival of cancer cells. Therefore, cell metabolism attracts the attention of researchers as a potential target. Here we demonstrate that TIM-4 enhances the OXPHOS level of lung cancer cells and the healthy status of mitochondria. As summarized in Fig. 7, TIM-4 enhances L-OPA1 levels by enhancing the activation of the ANXA2-PI3K/AKT axis, thereby promoting mitochondrial fusion. OPA1 is essential for the maintenance of mitochondrial kinetic balance and functional homeostasis. Our results reveal a novel mechanism by which TIM-4 promotes lung cancer proliferation and growth, identifying TIM-4 as a new regulator in mitochondria homeostasis of lung cancer cells.

It is well known that Warburg effect is the dominant metabolic pattern of most tumor cells. The promoting effect of Warburg effect on lung cancer progression has been reported [41, 42]. However, the metabolic pattern of tumor cells shows heterogeneity and plasticity, which has also been demonstrated in non-small cell lung cancer [43]. Emerging evidence indicates that some cancers are heavily dependent on OXPHOS [44]. Due to the metabolic heterogeneity of lung cancer, the tumor-promoting effect of OXPHOS cannot be ignored [45, 46]. The mitochondrial membrane potential (ΔΨm) is the main driver of OXPHOS [47]. The ability of mitochondria to respond to nutrient deficiencies can reflect the health status of mitochondria [28]. Our previous studies have shown that TIM-4 can promote lung cancer cell proliferation, but the mechanism remains to be further elucidated. In this study, we found that TIM-4 acts as a cancer-promoting factor to enhance OXPHOS in lung cancer cells. Meanwhile, we found that TIM-4 enhanced mitochondrial function, which was manifested as increased mitochondrial membrane potential, increased proportion of healthy mitochondria, increased adaptability to low-glucose conditions, and decreased ROS level. Regulation of mitochondrial function by TIM-4 has been reported in macrophages. TIM-4^+^ TAMs exhibit and maintain high mitochondria activity and autophagy function. Mechanically, arginase-1 impacts mitochondria fitness and mitophagy *via* mTORC1 in TIM-4^+^ TAMs [22]. Our results further reveal the mechanism by which TIM-4 regulates mitochondrial function. However, we did not examine whether TIM-4 also affects glycolysis in lung cancer cells, which requires to be further investigated in the future.

Mitochondria are strongly associated with carcinogenesis and mitochondrial dynamics balance plays an important role in the regulation of mitochondrial function and cell metabolism. Mitochondrial dynamic balance involves continuous processes of mitochondrial fusion by merging shorter mitochondrial fragments into larger and fission, which severs longer mitochondrial networks into smaller particles [48]. Mitochondrial fusion and fission are coordinated by multiple molecules, and the mitochondrial fusion-related molecule optic atrophy 1 (OPA1) locates in the inner mitochondrial membrane to integrate two membranes into one and also serves to maintain mitochondrial cristae structural fidelity [48]. Our results suggest that TIM-4 can maintain the protein level of L-OPA1, thereby promoting mitochondrial fusion in lung cancer cells, confirming that TIM-4 enhances OXPHOS in lung cancer cells by regulating mitochondrial dynamic balance. TIM-4 increased proliferation of lung cancer cells is significantly inhibited after interfere with siOPA1. A portion of L-OPA1 is hydrolyzed and cleaved outside the transmembrane segment by the intima anchoring protease OMA1 or YME1L, producing a slightly different non-membrane anchoring short form (S-OPA1) [49]. We don’t address whether TIM-4 has an effect on activity of OMA1 or YME1L, which would be an interesting problem to be solved. Unshown data indicate that TIM-4 overexpression can increase the number of mitochondria and upregulate the expression level of genes related to electron transport chain complex. Thus, we could not exclude that TIM-4 may also be involved in regulation of mitochondrial function in other ways in addition to the maintenance of mitochondrial kinetic balance through L-OPA1.

The PI3K/AKT signaling pathway is essential for the growth, proliferation, and survival of tumor cells. A large number of literatures have reported that the PI3K/AKT signaling pathway is involved in the growth [50], metastasis [51, 52], and therapeutic resistance [53] of lung cancer. As an important factor in cell metabolism regulation, the PI3K/AKT signaling pathway also plays an important role in mitochondrial function [54, 55]. The activation of PI3K/AKT signaling pathway is also regulated by a variety of molecules, including the activation of some growth factors and their receptors [56], and the activation of upstream key kinases such as PTEN [57]. TIM-4 enhances AKT activation, which has been reported in colon cancer and macrophages [20, 58], but the mechanism is unclear. In this study, we attempt to find the mechanism by which TIM-4 promotes PI3K/AKT activation. Our data show that TIM-4 interacts ANXA2 to activating AKT. Previously, ANXA2 has been reported to be involved in a variety of pathophysiological processes as a regulator of PI3K/AKT activation [39, 40, 59, 60]. Our results illustrate that ANXA2 is responsible for TIM-4-regulated PI3K/AKT activation. However, exact mechanism needs further verification.

In general, our study suggests that TIM-4 enhances cell proliferation by regulating mitochondrial function and homeostasis through the ANXA2/PI3K/AKT axis. We first identified the role of TIM-4 in promoting tumor development at the level of metabolic regulation, highlighting the new mechanism of TIM-4 promoting development and progression of lung cancer. As a membrane molecule, Tim-4 may become a new therapeutic target for tumor cell metabolic reprogramming.

## Materials and methods

### Cell lines

A549 and H23 cell lines were initially purchased from the Cell Bank of the Chinese Academy of Sciences (Shanghai, China). A549 cells were cultured in DMEM (C11995500CP, Gibco) and H23 cells were cultured in RPMI-1640 medium (C11875500BT, Gibco). A549 and H23 cell lines have been authenticated using short tandem repeat (STR) profiling. All cell lines were tested for the absence of mycoplasma contamination. All medium contained 10% fetal bovine serum (10270-106, Gibco) and 1% penicillin-streptomycin (p1400, Solarbio).

### Transfection and establishment of stable cell lines

For siRNA or plasmid transient transfection, cells were seeded in 6-well or 12-well plates and transfected with the expression vectors or specific siRNAs targeting OPA1 (5′-CCGGACCTTAGTGAATATAAA-3′), AKT1 (5′-GACCATGAACGAHTTTGAGTA-3′), or ANXA2 (5′-CGGGATGCTTTGAA-3′) using Lipo2000 reagent (11668019, Invitrogen). Stable lung cancer cell lines overexpressing TIM-4 were established. Briefly, A549 and H23 cell lines were infected with TIM-4 overexpression or control lentivirus followed by puromycin (2 μg/ml) selection for 2 weeks.

### Western blotting

Cell samples were collected and lysed in RIPA buffer (P0013D, Beyotime) with 1% PMSF (ST506, Beyotime) on ice for 30 min. Then lysates were heated at 95 °C for 5 min. Denatured samples (30 μg per lane) were separated by SDS-PAGE and transferred onto PVDF membrane. The membranes were incubated in blocking buffer 5% (w/v) Albumin Bovine V (A8850-5g, Solarbio) in Tris-buffered saline, 0.1% Tween 20 (TBS-T) for 1 h at room temperature (RT), then incubated with primary antibodies against TIM-4 (1:1000, HPA015625, Sigma), PCNA (1:1000, ab92552, Abcam), Cyclin A2 (1:5000, 18202-1-AP, Proteintech), Cyclin B1 (1:2000, 4138T, CST), β-ACTIN (1:5000, 66009-1-Ig, Proteintech), OPA1 (1:1000, ab42364, Abcam), MFN2 (1:1000, A12771, Abclonal), MFF (1:1000, A4874, Abclonal), DRP1 (1:1000, AP0812, Abclonal), Phospho-Akt (Ser473) (1:1000, 4058s, CST), AKT (1:1000, ab18785, CST), FLAG (1:5000, M185-3, MBL), ANXA2 (1:1000, ab41803, Abcam) and GAPDH (1:5000, 60004-1-Ig, Proteintech) for 16 h at 4 °C. After washing, the membranes were incubated with the secondary antibodies coupled to HRP for 60 min at RT. The blotting signals were visualized by chemiluminescence imaging system (Tanon-5200, Shanghai, China).

### ATP production assay

Cellular ATP levels were measured using ATP Determination Kit (S0026, Beyotime). Cells were collected and lysates were added. The supernatants were collected and ATP detection working solution was prepared for detection. The ATP standard curve was used to measure the ATP concentrations in cell samples, which was normalized by the protein concentration.

### Mitochondrial stress test

Oxygen consumption rate (OCR) was determined with a Seahorse XF96 Extracellular Flux Analyzer (Agilent Seahorse Bioscience) following protocols recommended by the manufacturers. Cells were seeded on XF96 cell culture plate (10,000 cells/well) except the background correction well. After sticking to the wall overnight under normal culture conditions, the cells were transferred to a non-CO2 incubator for 30 min with non-buffered assay medium (Agilent) before the assay. The baseline recordings were followed by sequential injection of 2 μM oligomycin, 2 μM FCCP, and 0.5 μM rotenone/antimycin A. Then, the XF Cell Mito Stress Test Kit (Agilent) was used for the assay.

### Mitochondrial function assay

MitoProbe JC-1 Assay Kit (M34152, Invitrogen) was used to detect mitochondrial membrane potential. Mitochondrial depolarization was detected by MitoTracker™ Green/Red (M7514, Invitrogen). Markers of healthy mitochondria were detected by MitoTracker™ Deep Red FM (M22426, Thermo). Mitochondrial ROS levels were detected by Mito Tracker Red CMXRos-special packaging (M7512, Invitrogen).

### RNA isolation and qRT-PCR

Cells were washed with ice-cold phosphate-buffered solution (PBS) and total RNA was extracted using TRNzol (DP404, Tiangen). 1 μg RNA was used for the reverse transcription. cDNA was diluted 1:10 and used for qPCR with qPCR mix (FP205-02, Vazyme). Expression levels of mRNA were normalized to β-actin. The following oligonucleotides were used for analysis: human β-actin (Forward: 5′-ACATCCGCAAAGACC TGTACG-3′, Reverse: 5′-TTGCTGATCCACATCTGCTGG-3′); human OPA1(Forward: 5′-GTGGTTGGAGATCAGAGTGCTG-3′, Reverse: 5′-GAGGACCTTCACTCAGAGTCAC-3′); human ANXA2 (Forward: 5′-TCGGACACATCTGGTGACTTCC-3′, Reverse: 5′-CCTCTTCACTCCAGCGTCATAG-3′).

### Immunofluorescence staining and confocal laser scanning

A549 or H23 cells were seeded onto microscope cover glass in 24-well plates overnight. Cells were fixed in 4% fresh paraformaldehyde (PFA) for 30 min, then permeated with 0.2% Triton X-100, and blocked with bovine serum albumin for 30 min. After blocking, cells were incubated with the primary antibodies at 4 °C for 16 h, followed by incubation with the secondary antibodies conjugated with Alexa fluor-488 and Alexa fluor-594 (1:200) at RT for 1 h. Nuclei were stained with hochest 33342 for 5 min. After antibody incubation, the coverslips were washed thoroughly with tris-buffered solution (TBS) and mounted with fluoromount (ab104135, Abcam). Images were captured using the laser confocal microscope (LSM880, Germany) and ZEN 2.3 blue edition (Zeiss).

### Flow cytometry analysis

A549 or H23 cells were seeded on 24-well plates and labeled with JC-1 mitochondrial membrane potential assay kit (M34152, Invitrogen), MitoTracker™ Deep Red (M22426, Invitrogen), MitoTracker™ Green (M7514, Invitrogen), MitoTracker™ Red (M7512, Invitrogen) for 20 min at 37 °C. Flow cytometry assay was performed on a Cytoflex S (Beckman Coulter) or Gallios flow cytometer and analyzed by FlowJo 10.6.2 or CytExpert 2.3.0.

Intracellular staining for Ki67 was performed with the Fixation/Permeabilization Concentrate and Diluent Buffer Set according to the manufacturer’s guidelines (eBioscience). Then anti-Ki67-BV421 (350506, BioLegend) staining was performed for 1 h at 4 °C in the dark.

### RNA sequencing

A549-LV-TIM-4 and corresponding control cells were harvested and RNA sequencing (RNA-seq) was carried out by the Beijing Genomics Institute, following standard protocols. Essentially, differential expression analysis was performed using the PoissonDis algorithm with false discovery rate (FDR) ≤ 0.001 and |Log2Ratio| ≥ 1. To gain insight into the change of phenotype, GO (http://www.geneontology.org/) and Kyoto Encyclopedia of Gene and Genomes (https://www.kegg.jp/) enrichment analysis of annotated differently expressed genes was performed by phyper (https://stat.ethz.ch/R-manual/R-devel/library/stats/html/Hypergeometric.html) on the basis of a hypergeometric test. The significance levels of terms and pathways were corrected by Q value with a rigorous threshold (Q ≤ 0.05) by the Bonferroni correction.

### Co-immunoprecipitation

Cell lysates were incubated with anti-Flag or anti-ANXA2 antibodies and rotated overnight at 4 ^◦^C, followed by incubation with protein A/G magnetic beads for 4 h. Subsequently, the complex was pelleted and washed with PBST buffer five times. The pellet was suspended in 1 × SDS sample buffer, boiled, and subjected to SDS-PAGE for western blotting analysis.

### Statistical analysis

Sample sizes were denoted in the figure legends. All experiments were performed in triplicate. Samples included in the analyses surely met proper experimental conditions. All statistical analysis was performed using GraphPad Prism 8.0.1 software. Results are expressed as mean ± SEM from three or more independent experiments to ensure adequate power (>80%). Unpaired Student’s *t* test was performed to determine the statistical significance of differences between groups. Significance levels are indicated by asterisks: ***P < 0.001; **P < 0.01; *P < 0.05; ns means non-significance.

## Supplementary information

Supplementary Materials for TIM-4 orchestrates mitochondrial homeostasis to promote lung cancer progression via ANXA2/PI3K/AKT/OPA1 axis

Supplementary figure 1

Supplementary figure 2

Supplementary figure 3

Supplementary figure 4

Original Data File

## Data Availability

All data are available in the main text or the supplementary materials. The original contributions presented in the study are included in the article/Supplementary Material. The RNA-sequencing data (PRJNA932173) for this study are available in the Sequence Read Archive (SRA) database. Further inquiries can be directed to the corresponding author.

## References

[1] MacDonagh L, Gray SG, Breen E, Cuffe S, Finn SP, O’Byrne KJ (2016). Lung cancer stem cells: the root of resistance. Cancer Lett.

[2] Leon G, MacDonagh L, Finn SP, Cuffe S, Barr MP (2016). Cancer stem cells in drug resistant lung cancer: targeting cell surface markers and signaling pathways. Pharm Ther.

[3] Hanahan D, Weinberg RA (2011). Hallmarks of cancer: the next generation. Cell.

[4] Anastasiou D (2017). Tumour microenvironment factors shaping the cancer metabolism landscape. Br J Cancer.

[5] Greene J, Segaran A, Lord S (2022). Targeting OXPHOS and the electronic transport chain in cancer; molecular and therapeutic implications. Semin Cancer Biol.

[6] Warburg O. On the origin of cancer cells. Science. 1956;123:309–14. 10.1126/science.123.3191.309 13298683

[7] Warburg O, Wind F, Negelein E. The metabolism of tumors in the body. J Gen Physiol. 1927;8:519. 10.1085/jgp.8.6.519 PMC2140820 19872213

[8] Lu J, Tan M, Cai Q (2015). The Warburg effect in tumor progression: mitochondrial oxidative metabolism as an anti-metastasis mechanism. Cancer Lett.

[9] Danhier P, Banski P, Payen VL, Grasso D, Ippolito L, Sonveaux P (2017). Cancer metabolism in space and time: Beyond the Warburg effect. Biochim Biophys Acta Bioenerg.

[10] Zong WX, Rabinowitz JD, White E (2016). Mitochondria and cancer. Mol Cell.

[11] Wu Z, Zuo M, Zeng L, Cui K, Liu B, Yan C (2021). OMA1 reprograms metabolism under hypoxia to promote colorectal cancer development. EMBO Rep.

[12] Zampieri LX, Silva-Almeida C, Rondeau JD, Sonveaux P (2021). Mitochondrial transfer in cancer: a comprehensive review. Int J Mol Sci.

[13] Liu L, Zhang X, Ding H, Liu X, Cao D, Liu Y (2021). Arginine and lysine methylation of MRPS23 promotes breast cancer metastasis through regulating OXPHOS. Oncogene.

[14] Kumar PR, Moore JA, Bowles KM, Rushworth SA, Moncrieff MD (2021). Mitochondrial oxidative phosphorylation in cutaneous melanoma. Br J Cancer.

[15] Freeman GJ, Casasnovas JM, Umetsu DT, DeKruyff RH. *TIM* genes: a family of cell surface phosphatidylserine receptors that regulate innate and adaptive immunity. Immunol Rev. 2010;235:172–89. 10.1111/j.0105-2896.2010.00903.x PMC2914464 20536563

[16] Kobayashi N, Karisola P, Pena-Cruz V, Dorfman DM, Jinushi M, Umetsu SE (2007). T cell immunoglobulin mucin protein (TIM)-4 binds phosphatidylserine and mediates uptake of apoptotic cells. Immunity.

[17] Tietjen GT, Gong Z, Chen CH, Vargas E, Crooks JE, Cao KD (2014). Molecular mechanism for differential recognition of membrane phosphatidylserine by the immune regulatory receptor Tim4. Proc Natl Acad Sci USA.

[18] Chow A, Schad S, Green MD, Hellmann MD, Allaj V, Ceglia N (2021). Tim-4(+) cavity-resident macrophages impair anti-tumor CD8(+) T cell immunity. Cancer Cell.

[19] Zhang Q, Wang H, Wu X, Liu B, Liu W, Wang R (2015). TIM-4 promotes the growth of non-small-cell lung cancer in a RGD motif-dependent manner. Br J Cancer.

[20] Tan X, Zhang Z, Yao H, Shen L (2018). Tim-4 promotes the growth of colorectal cancer by activating angiogenesis and recruiting tumor-associated macrophages via the PI3K/AKT/mTOR signaling pathway. Cancer Lett.

[21] Liu W, Wang H, Bai F, Ding L, Huang Y, Lu C (2020). IL-6 promotes metastasis of non-small-cell lung cancer by up-regulating TIM-4 via NF-kappaB. Cell Prolif.

[22] Xia H, Li S, Li X, Wang W, Bian Y, Wei S (2020). Autophagic adaptation to oxidative stress alters peritoneal residential macrophage survival and ovarian cancer metastasis. JCI Insight.

[23] Bennett CF, O’Malley KE, Perry EA, Balsa E, Latorre-Muro P, Riley CL (2021). Peroxisomal-derived ether phospholipids link nucleotides to respirasome assembly. Nat Chem Biol.

[24] Fernandez-Vizarra E, Zeviani M (2021). Mitochondrial disorders of the OXPHOS system. FEBS Lett.

[25] Kosaisawe N, Sparta B, Pargett M, Teragawa CK, Albeck JG (2021). Transient phases of OXPHOS inhibitor resistance reveal underlying metabolic heterogeneity in single cells. Cell Metab.

[26] Swadling L, Pallett LJ, Diniz MO, Baker JM, Amin OE, Stegmann KA (2020). Human liver memory CD8(+) T cells use autophagy for tissue residence. Cell Rep.

[27] Dan Dunn J, Alvarez LA, Zhang X, Soldati T (2015). Reactive oxygen species and mitochondria: a nexus of cellular homeostasis. Redox Biol.

[28] Lin S, Huang C, Gunda V, Sun J, Chellappan SP, Li Z (2019). Fascin controls metastatic colonization and mitochondrial oxidative phosphorylation by remodeling mitochondrial actin filaments. Cell Rep.

[29] Birsoy K, Wang T, Chen WW, Freinkman E, Abu-Remaileh M, Sabatini DM (2015). An essential role of the mitochondrial electron transport chain in cell proliferation is to enable aspartate synthesis. Cell.

[30] Weiner-Gorzel K, Murphy M (2021). Mitochondrial dynamics, a new therapeutic target for Triple Negative Breast Cancer. Biochim Biophys Acta Rev Cancer.

[31] Frezza C, Cipolat S, Martins de Brito O, Micaroni M, Beznoussenko GV, Rudka T (2006). OPA1 controls apoptotic cristae remodeling independently from mitochondrial fusion. Cell.

[32] Herkenne S, Ek O, Zamberlan M, Pellattiero A, Chergova M, Chivite I (2020). Developmental and tumor angiogenesis requires the mitochondria-shaping protein Opa1. Cell Metab.

[33] Alzahrani AS (2019). PI3K/Akt/mTOR inhibitors in cancer: at the bench and bedside. Semin Cancer Biol.

[34] Lee CM, Fuhrman CB, Planelles V, Peltier MR, Gaffney DK, Soisson AP (2006). Phosphatidylinositol 3-kinase inhibition by LY294002 radiosensitizes human cervical cancer cell lines. Clin Cancer Res.

[35] Weinman JS, Feinberg JM, Rainteau DP, Gaspera BD, Weinman SJ. Annexins in rat enterocyte and hepatocyte an immunogold electron-microscope study. Cell Tissue Res. 1994;278:389–97. 10.1007/BF00414181 8001090

[36] Diakonova M, Gerke V, Ernst JP, Liautard JP, van der Vusse G, Griffiths G. Localization of five annexins in J774 macrophages and on isolated phagosomes. J Cell Sci. 1997;110:1199–213. 10.1242/jcs.110.10.1199 9191044

[37] Anselmino N, Bizzotto J, Sanchis P, Lage-Vickers S, Ortiz E, Valacco P (2020). HO-1 interactors involved in the colonization of the bone niche: role of ANXA2 in prostate cancer progression. Biomolecules.

[38] Buttarelli M, Babini G, Raspaglio G, Filippetti F, Battaglia A, Ciucci A (2019). A combined ANXA2-NDRG1-STAT1 gene signature predicts response to chemoradiotherapy in cervical cancer. J Exp Clin Cancer Res.

[39] Castaldo SA, Ajime T, Serrao G, Anastacio F, Rosa JT, Giacomantonio CA (2019). Annexin A2 regulates AKT upon H(2)O(2)-dependent signaling activation in cancer cells. Cancers.

[40] Chaudhary P, Thamake SI, Shetty P, Vishwanatha JK (2014). Inhibition of triple-negative and Herceptin-resistant breast cancer cell proliferation and migration by Annexin A2 antibodies. Br J Cancer.

[41] Xue L, Li J, Lin Y, Liu D, Yang Q, Jian J (2021). m(6) A transferase METTL3-induced lncRNA ABHD11-AS1 promotes the Warburg effect of non-small-cell lung cancer. J Cell Physiol.

[42] Xie M, Fu XG, Jiang K (2021). Notch1/TAZ axis promotes aerobic glycolysis and immune escape in lung cancer. Cell Death Dis.

[43] Hensley CT, Faubert B, Yuan Q, Lev-Cohain N, Jin E, Kim J (2016). Metabolic heterogeneity in human lung tumors. Cell.

[44] Oliveira GL, Coelho AR, Marques R, Oliveira PJ (2021). Cancer cell metabolism: rewiring the mitochondrial hub. Biochim Biophys Acta Mol Basis Dis.

[45] Rao S, Mondragon L, Pranjic B, Hanada T, Stoll G, Kocher T (2019). AIF-regulated oxidative phosphorylation supports lung cancer development. Cell Res.

[46] Kalainayakan SP, FitzGerald KE, Konduri PC, Vidal C, Zhang L (2018). Essential roles of mitochondrial and heme function in lung cancer bioenergetics and tumorigenesis. Cell Biosci.

[47] Wolf DM, Segawa M, Kondadi AK, Anand R, Bailey ST, Reichert AS (2019). Individual cristae within the same mitochondrion display different membrane potentials and are functionally independent. EMBO J.

[48] Sessions DT, Kashatus DF (2021). Mitochondrial dynamics in cancer stem cells. Cell Mol Life Sci.

[49] Wang R, Mishra P, Garbis SD, Moradian A, Sweredoski MJ, Chan DC (2021). Identification of new OPA1 cleavage site reveals that short isoforms regulate mitochondrial fusion. Mol Biol Cell.

[50] Yu X, Li Y, Jiang G, Fang J, You Z, Shao G (2021). FGF21 promotes non-small cell lung cancer progression by SIRT1/PI3K/AKT signaling. Life Sci.

[51] Wei C, Dong X, Lu H, Tong F, Chen L, Zhang R (2019). LPCAT1 promotes brain metastasis of lung adenocarcinoma by up-regulating PI3K/AKT/MYC pathway. J Exp Clin Cancer Res.

[52] Liang J, Li H, Han J, Jiang J, Wang J, Li Y (2020). Mex3a interacts with LAMA2 to promote lung adenocarcinoma metastasis via PI3K/AKT pathway. Cell Death Dis.

[53] Jin Y, Chen Y, Tang H, Hu X, Hubert SM, Li Q (2022). Activation of PI3K/AKT pathway is a potential mechanism of treatment resistance in small cell lung cancer. Clin Cancer Res.

[54] Zheng X, Li W, Xu H, Liu J, Ren L, Yang Y (2021). Sinomenine ester derivative inhibits glioblastoma by inducing mitochondria-dependent apoptosis and autophagy by PI3K/AKT/mTOR and AMPK/mTOR pathway. Acta Pharm Sin B.

[55] Stiles BL (2009). PI-3-K and AKT: onto the mitochondria. Adv Drug Deliv Rev.

[56] Molinaro A, Becattini B, Mazzoli A, Bleve A, Radici L, Maxvall I (2019). Insulin-driven PI3K-AKT signaling in the hepatocyte is mediated by redundant PI3Kalpha and PI3Kbeta activities and is promoted by RAS. Cell Metab.

[57] Gehringer F, Weissinger SE, Moller P, Wirth T, Ushmorov A (2020). Physiological levels of the PTEN-PI3K-AKT axis activity are required for maintenance of Burkitt lymphoma. Leukemia.

[58] Wu H, Chen G, Wang J, Deng M, Yuan F, Gong J (2020). TIM-4 interference in Kupffer cells against CCL4-induced liver fibrosis by mediating Akt1/Mitophagy signalling pathway. Cell Prolif.

[59] Pan H (2021). Radiation engenders converse migration and invasion in colorectal cancer cells through opposite modulation of__ANXA2_AKT_GSK3β pathway. Am J Cancer Res.

[60] Zhao C, Zheng S, Yan Z, Deng Z, Wang R, Zhang B (2020). CCL18 promotes the invasion and metastasis of breast cancer through Annexin A2. Oncol Rep.

