# Supplementary material for: TIM-4 orchestrates mitochondrial homeostasis to promote lung cancer progression via ANXA2/PI3K/AKT/OPA1 axis
Source: Cell Death Dis. 2023 Feb 20;14(2):141. doi: 10.1038/s41419-023-05678-3 (PMC9941510; doi:10.1038/s41419-023-05678-3)
Supplement: Supplementary file 1 — Supplementary Materials for TIM-4 orchestrates mitochondrial homeostasis to promote lung cancer progression via ANXA2/PI3K/AKT/OPA1 axis [file 41419_2023_5678_MOESM1_ESM.docx]

Supplementary Materials for

# TIM-4 orchestrates mitochondrial homeostasis to promote lung cancer progression *via* ANXA2/PI3K/AKT/OPA1 axis

Yuzhen Wang^1^, Yingchun Wang^1^, Wen Liu^1^, Lu Ding^1^, Xiaodi Zhang^1^, Bo Wang^1^, Zheng Tong^1^, Liyun Xu^4^, Xuetian Yue^2^, Chunyang Li^3^, Zhuanchang Wu^1^, Xiaohong Liang^1^, Chunhong Ma^1^, Lifen Gao^1^* ([glfflg@sdu.edu.cn.](mailto:glfflg@sdu.edu.cn))

# This file includes: Supplementary Figure Legends. S1 to S4

**Fig. S1 TIM-4 enhances the oxidative phosphorylation level of lung cancer cells. A** Fluorescence expression in lung cancer cell lines after transfected with LV-CON or LV- TIM-4 (Scale bar:100 μm). **B** Efficiency of TIM-4 expression was analyzed by western blotting. **C** Visualization of correlation coefficient matrix of RNA sequencing in A549 cells. **D and E** O2K respirometer system was utilized to determine the oxygen consumption level of A549 cells after overexpression of TIM-4

**Fig. S2 TIM-4 promotes mitochondrial function and activity via L-OPA1.**

**A and B** RT-qPCR showed the expression changes of OPA1 in A549 and H23 cells. **C** Representative TEM images of mitochondria in H23 cells. (Bar = 1.0 μm). **D** OPA1 levels were assessed by immunoblotting in lung cancer cell lines transfected with siRNA targeting OPA1. **E and F** siRNA targeting OPA1 were transfected in A549 and H23 cells, then OCR, mitochondrial basal respiration and maximal respiration were tested. siRNA targeting OPA1 was transfected into A549 and H23 cells, and then mitochondrial function and activity were tested. siRNA targeting OPA1 was transfected into A549 and H23 cells, and then mitochondrial function and activity were tested. **G and H** Mitochondrial membrane potential (JC-1 aggregates) in A549 cells and H23 cells were accessed by the JC-1 assay kit according to the manufacture’s instruction. **I and J** Flow cytometry dot plots showed the percentage of depolarized mitochondria in A549 and H23 cells transfected with LV-CON or LV-TIM-4. **K and L** Mitochondria fitness was tested with Mito-tracker deep red. Three independent experiments were conducted for each result and error bars represent SEM per group in one experiment. Data were analyzed using Student’s *t* test (two-tailed paired *t* test). ns means non significance; **, P < 0.05; **, P < 0.01; ***, P < 0.001.*

**Fig. S3** **Interference with AKT1 expression eliminates the effect of TIM-4 on mitochondrial homeostasis in lung cancer cells. A** Immunoblotting was used to determine the interference efficiency of siAKT1 in A549 and H23 cells. **B and C** siRNA targeting AKT1 was transfected into A549 and H23 cells, and then OCR, mitochondrial basal respiration and maximal respiration were tested. **D and E** Mitochondrial membrane potential (JC-1 aggregates) in A549 cells and H23 cells were accessed by the JC-1 assay kit according to the manufacture’s instruction. **F and G** Flow cytometry dot plots showed the percentage of depolarized mitochondria in A549 and H23 cells transfected with LV-CON or LV-TIM-4. **H and I** Mitochondria fitness was tested with Mito-tracker deep red. Three independent experiments were conducted for each result and error bars represent SEM per group in one experiment. Data were analyzed using Student’s *t* test (two-tailed paired *t* test). ns means non significance; **, P < 0.05; **, P < 0.01; ***, P < 0.001.*

**Fig. S4 TIM-4 interacts with ANXA2. A** Overlapping proteins in A549 and HEK-293 cells. **B** RT-qPCR showed the expression changes of ANXA2 in A549 and H23 cells. Three independent experiments were conducted for each result and error bars represent SEM per group in one experiment. Data were analyzed using Student’s *t* test (two-tailed paired *t* test). ns means non significance; **, P < 0.05; **, P < 0.01; ***, P < 0.001*.
