# Supplementary figures and images for: TIM-4 orchestrates mitochondrial homeostasis to promote lung cancer progression via ANXA2/PI3K/AKT/OPA1 axis
Source: Cell Death Dis. 2023 Feb 20;14(2):141. doi: 10.1038/s41419-023-05678-3 (PMC9941510; doi:10.1038/s41419-023-05678-3)

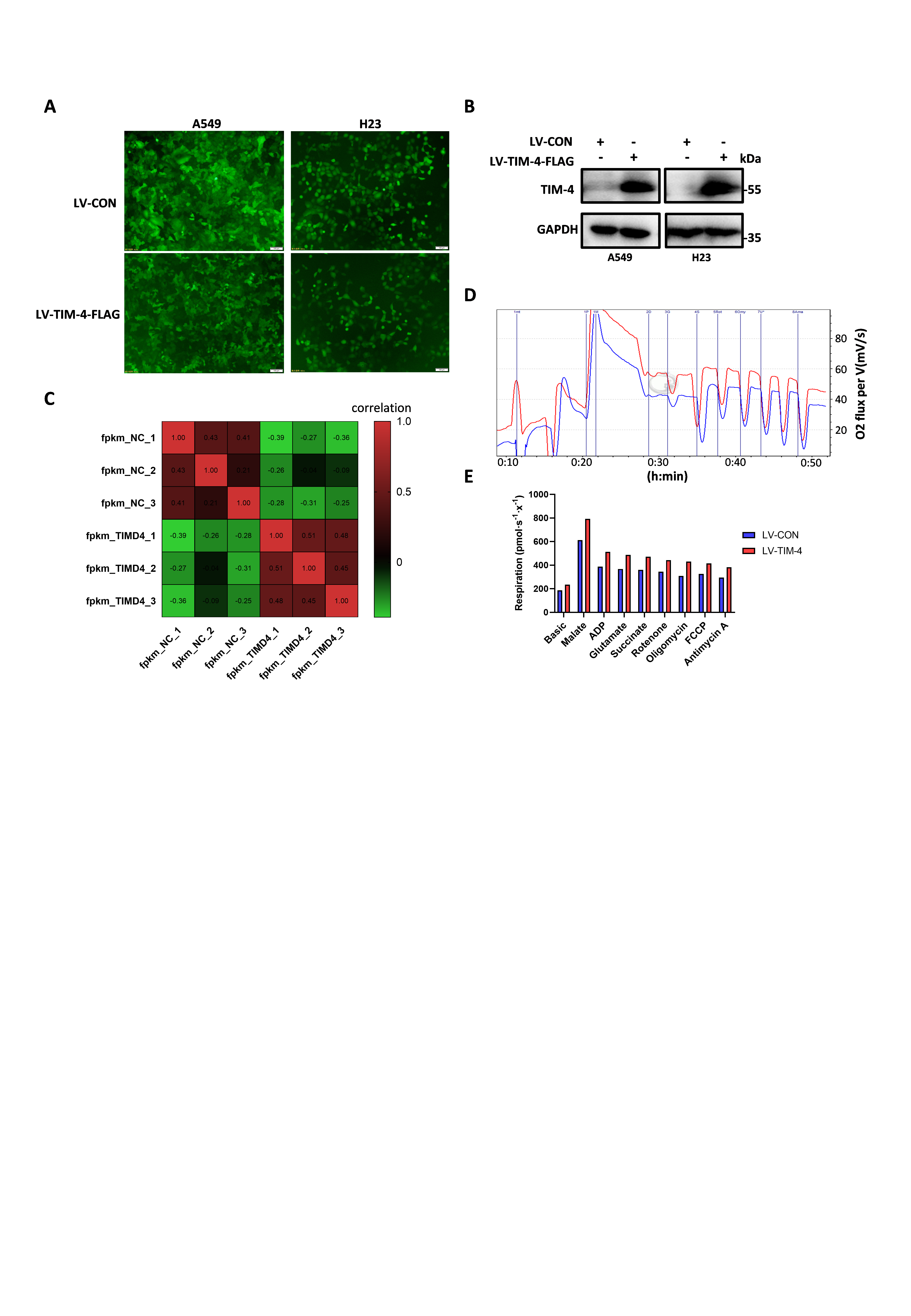

Supplement: Supplementary file 2 — Supplementary figure 1 [file 41419_2023_5678_MOESM2_ESM.tif]

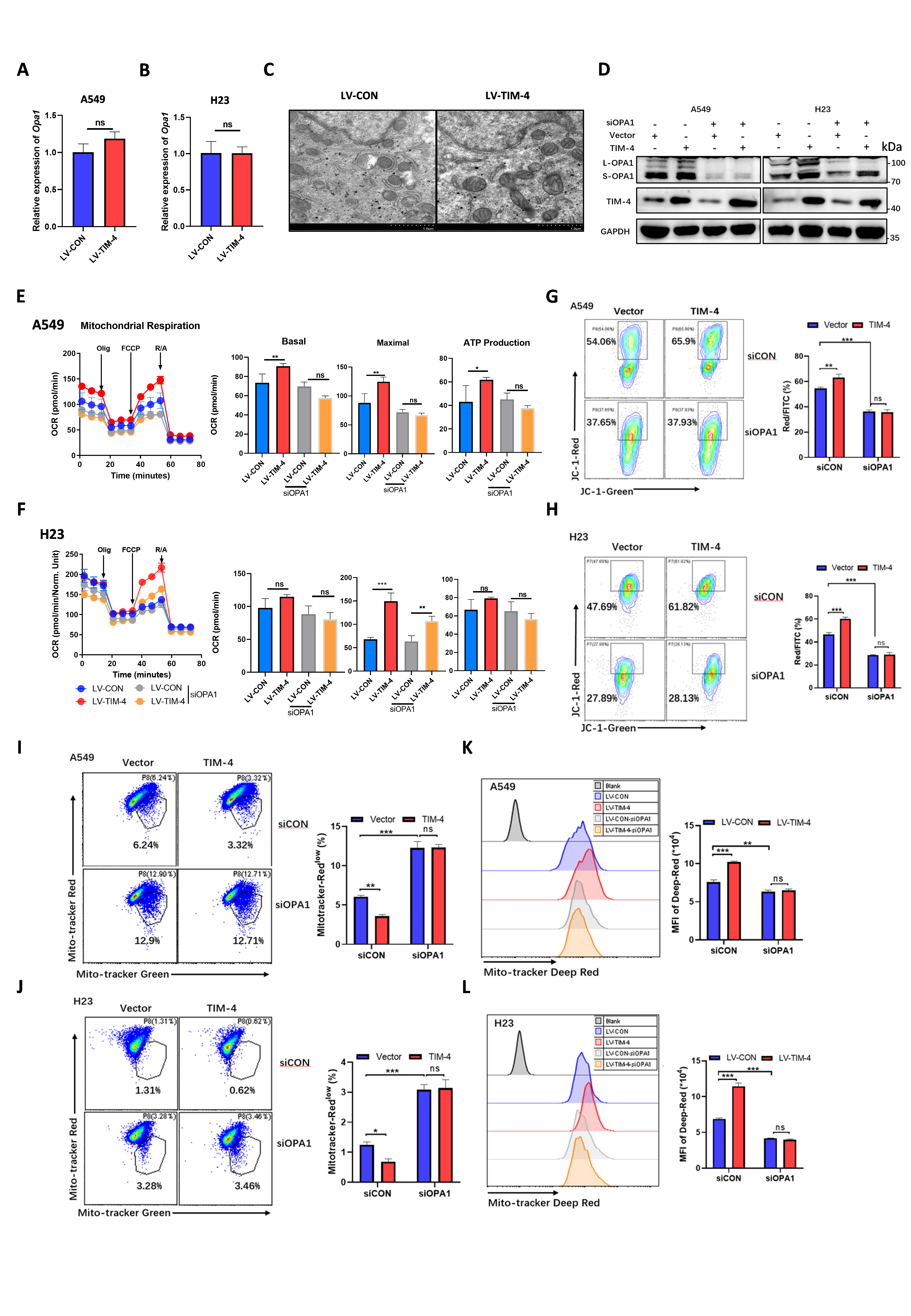

Supplement: Supplementary file 3 — Supplementary figure 2 [file 41419_2023_5678_MOESM3_ESM.tif]

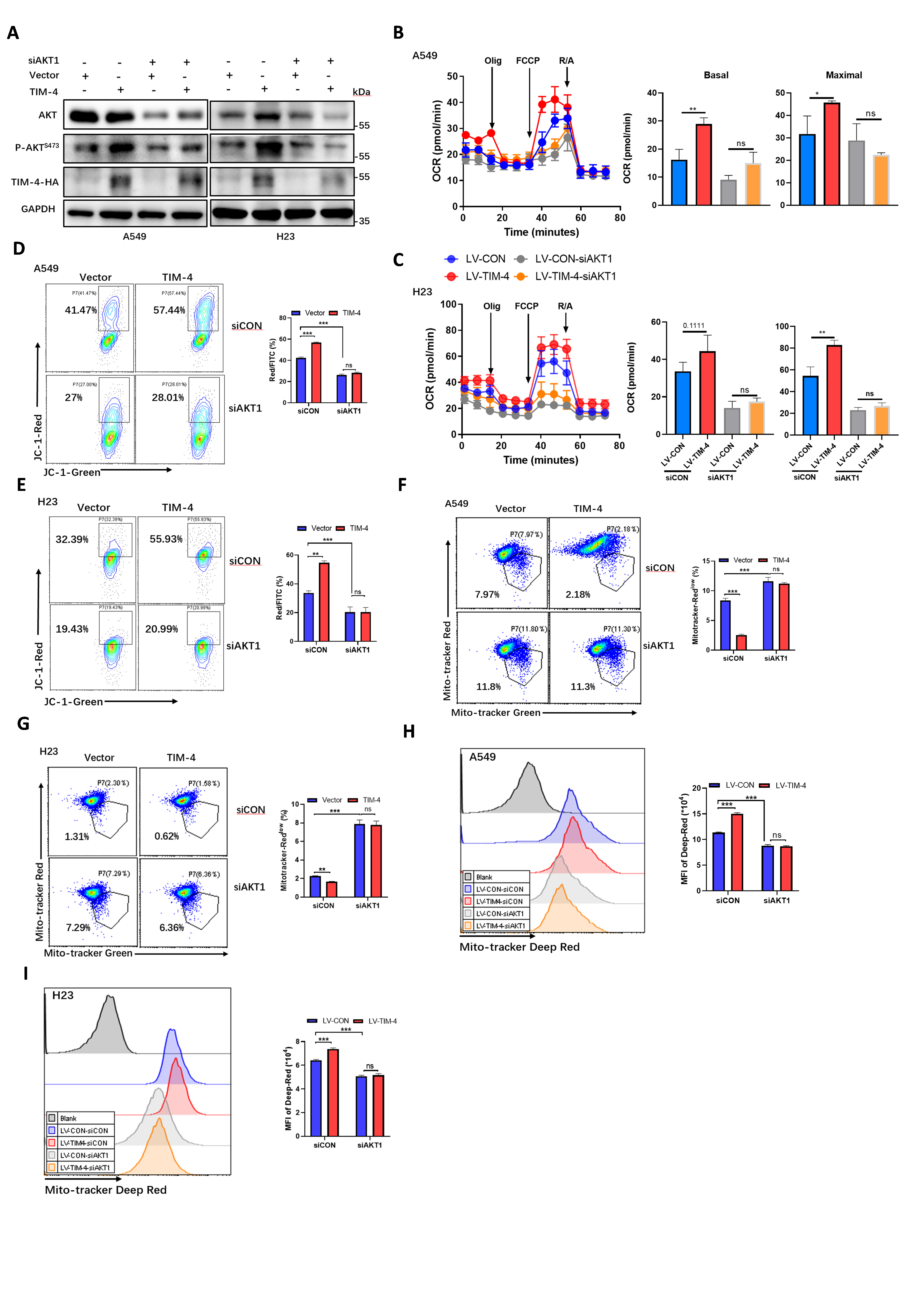

Supplement: Supplementary file 4 — Supplementary figure 3 [file 41419_2023_5678_MOESM4_ESM.tif]

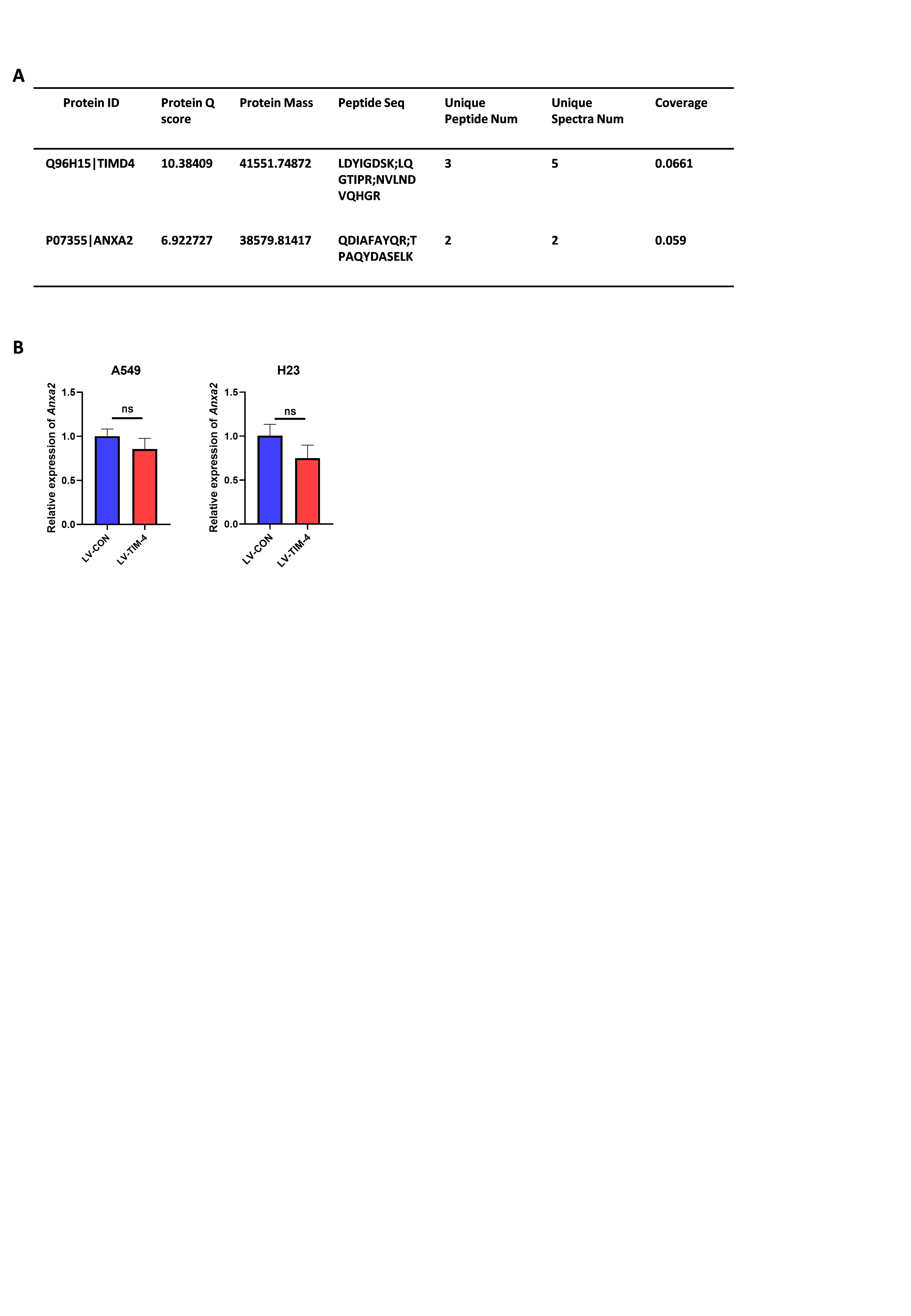

Supplement: Supplementary file 5 — Supplementary figure 4 [file 41419_2023_5678_MOESM5_ESM.tif]

**Original western blots**


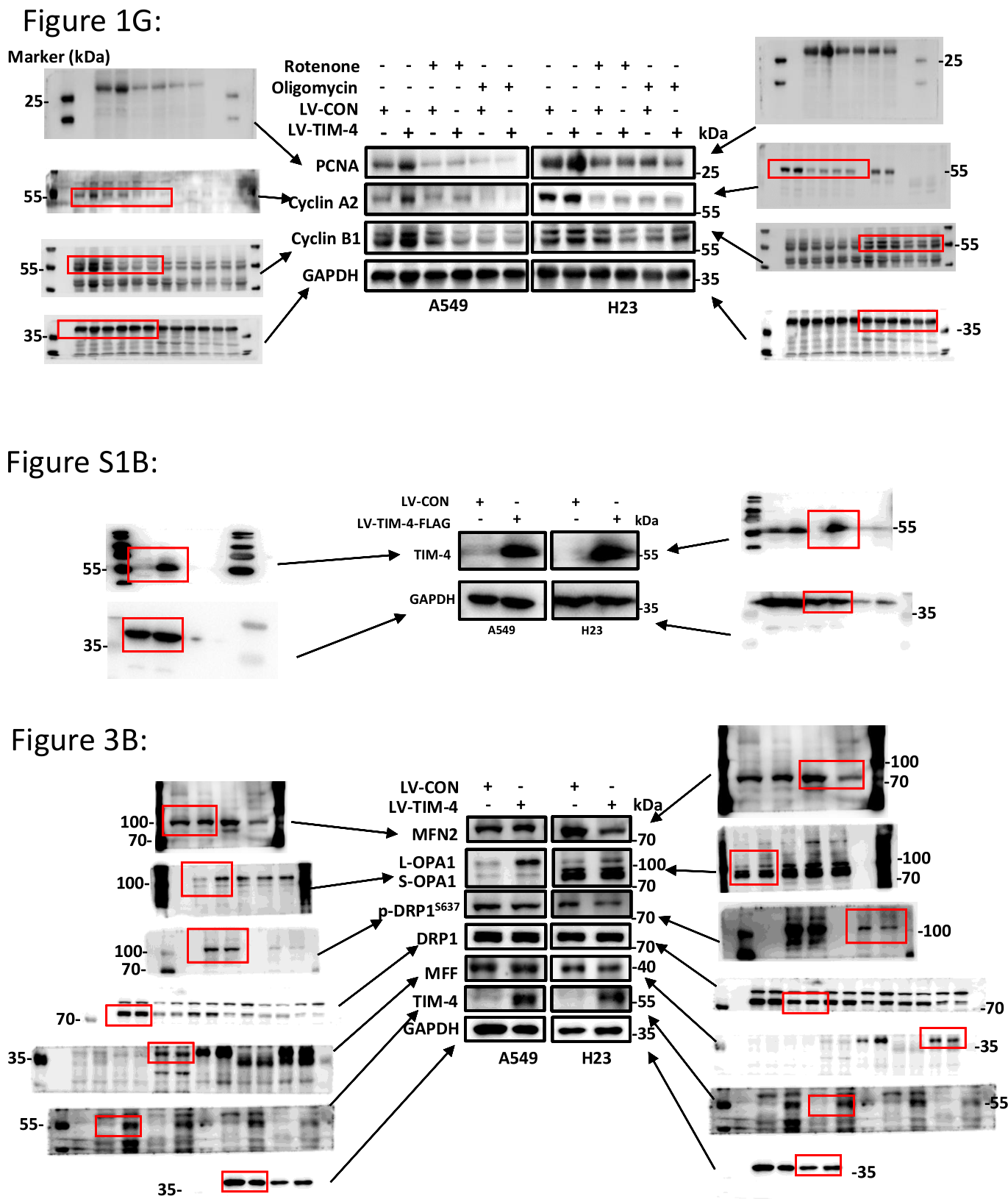

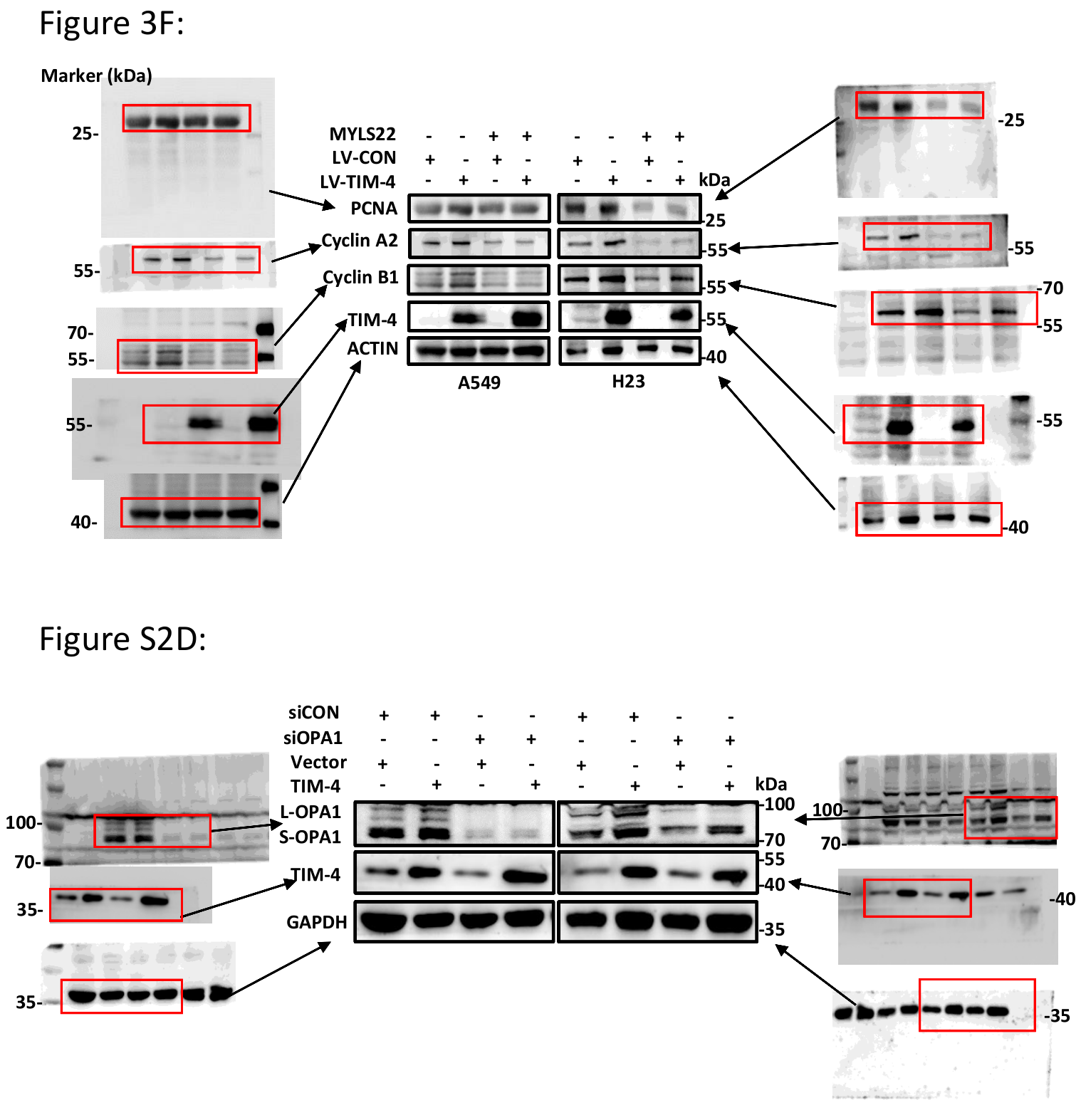

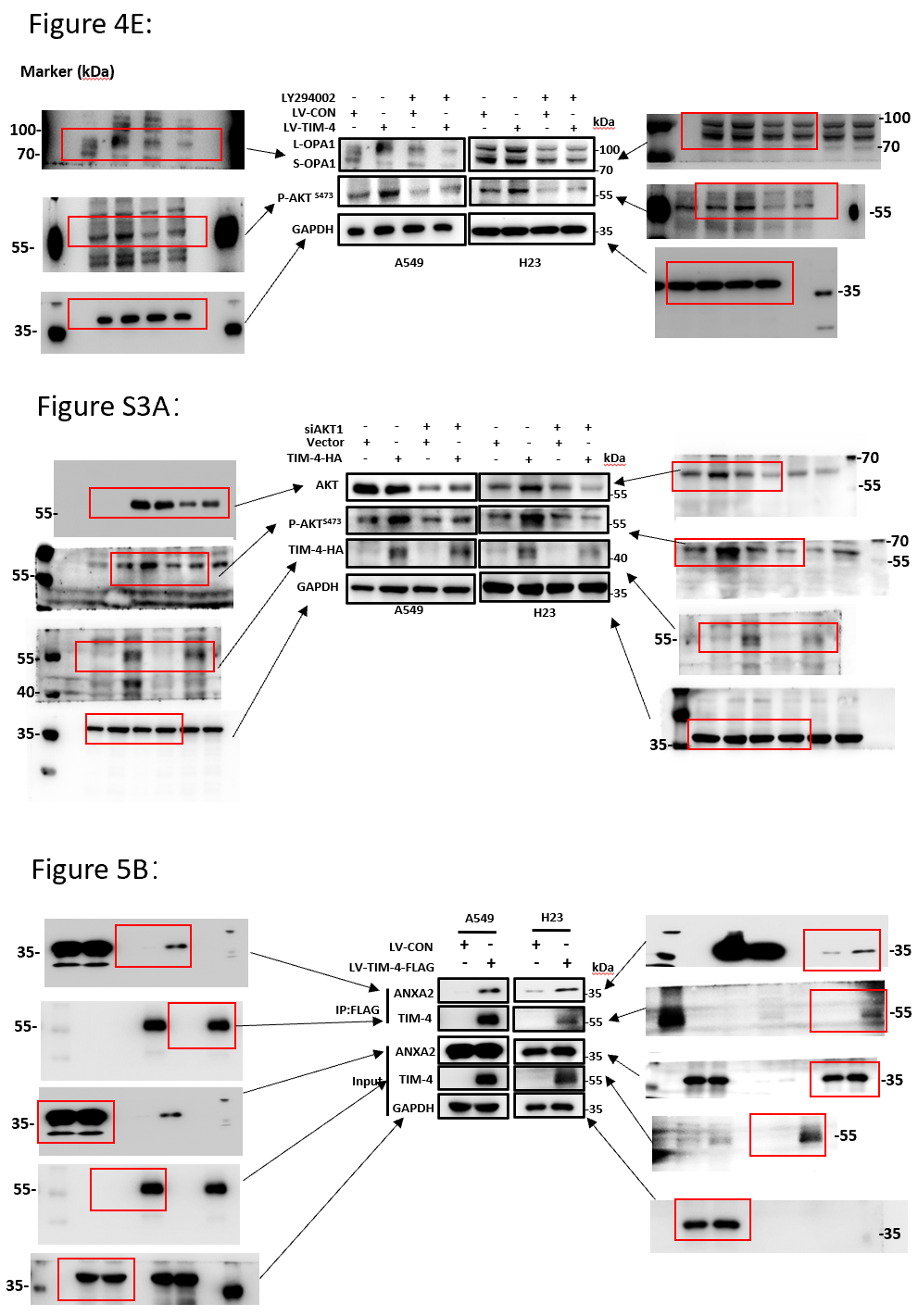


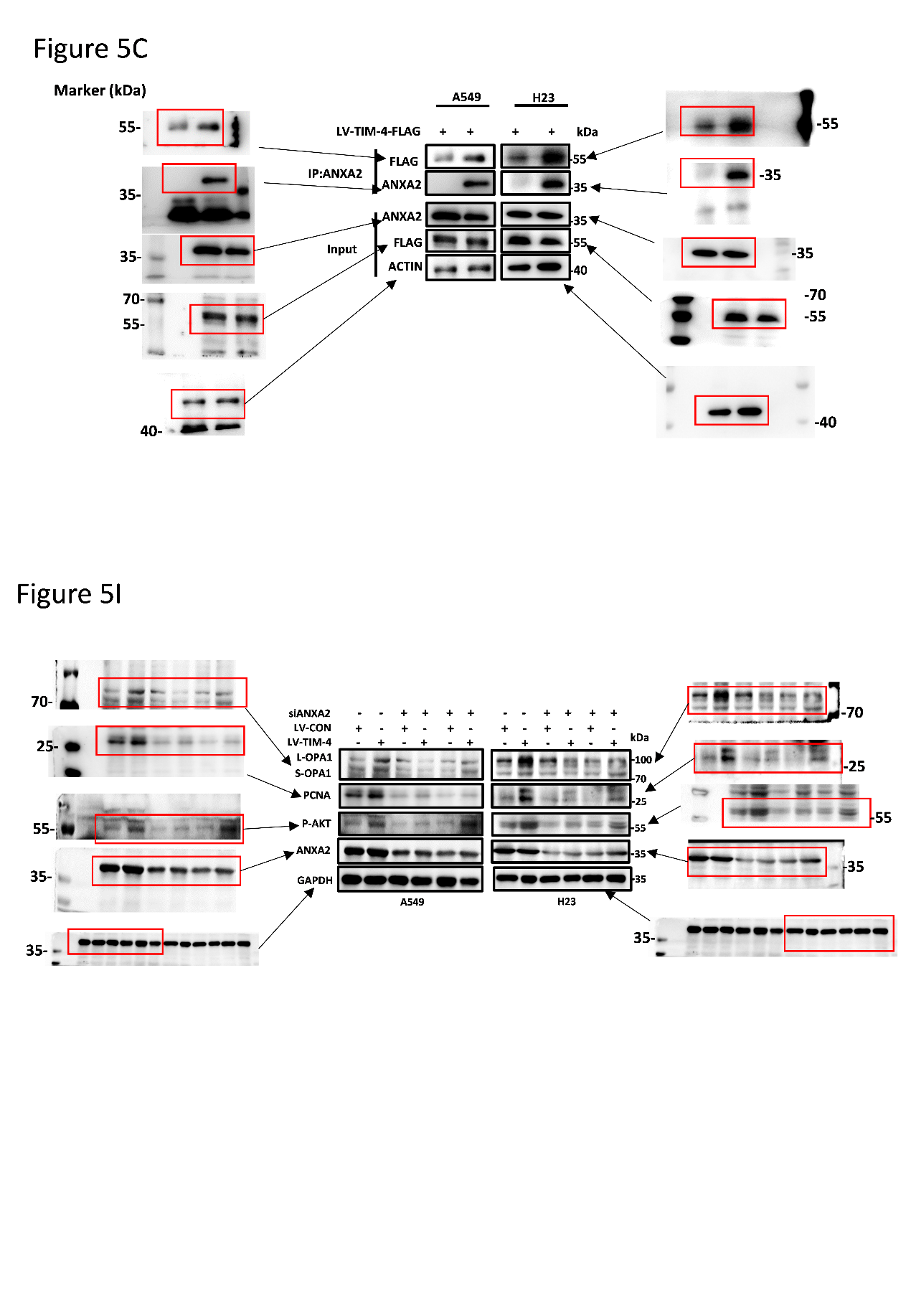

Supplement: Supplementary file 6 — Original Data File [file 41419_2023_5678_MOESM6_ESM.docx]
